# Alcohol Dependence Modulates Amygdalar mTORC2 and PKCε Expression in a Rodent Model

**DOI:** 10.3390/nu15133036

**Published:** 2023-07-05

**Authors:** Athirah Hanim, Isa N. Mohamed, Rashidi M. P. Mohamed, Mohd Helmy Mokhtar, Suzana Makpol, Ruth Naomi, Hasnah Bahari, Haziq Kamal, Jaya Kumar

**Affiliations:** 1 Department of Physiology, Faculty of Medicine, Universiti Kebangsaan Malaysia, Cheras, Kuala Lumpur 56000, Malaysia; trahtyrah1990@gmail.com (A.H.); helmy@ukm.edu.my (M.H.M.); kamalntee1@gmail.com (H.K.); 2 Department of Pharmacology, Faculty of Medicine, Universiti Kebangsaan Malaysia, Cheras, Kuala Lumpur 56000, Malaysia; isanaina@ppukm.ukm.edu.my; 3 Department of Family Medicine, Faculty of Medicine, Universiti Kebangsaan Malaysia, Cheras, Kuala Lumpur 56000, Malaysia; drrashidi5377@yahoo.com.my; 4 Department of Biochemistry, Faculty of Medicine, Universiti Kebangsaan Malaysia, Cheras, Kuala Lumpur 56000, Malaysia; suzanamakpol@ppukm.ukm.edu.my; 5 Department of Human Anatomy, Faculty of Medicine and Health Sciences, Universiti Putra Malaysia, Serdang 43400, Malaysia; gs60018@student.upm.edu.my (R.N.); haba@upm.edu.my (H.B.)

**Keywords:** alcohol, amygdala, mTOR, mTORC2, mTORC1, PKC epsilon, alcohol dependence

## Abstract

Multiple alcohol use disorder (AUD)-related behavioral alterations are governed by protein kinase C epsilon (PKCε), particularly in the amygdala. Protein kinase C (PKC) is readily phosphorylated at Ser729 before activation by the mTORC2 protein complex. In keeping with this, the current study was conducted to assess the variations in mTORC2 and PKCε during different ethanol exposure stages. The following groups of rats were employed: control, acute, chronic, ethanol withdrawal (EW), and EW + ethanol (EtOH). Ethanol-containing and non-ethanol-containing modified liquid diets (MLDs) were administered for 27 days. On day 28, either saline or ethanol (2.5 g/kg, 20% *v*/*v*) was intraperitoneally administered, followed by bilateral amygdala extraction. PKCε mRNA levels were noticeably increased in the amygdala of the EW + EtOH and EW groups. Following chronic ethanol consumption, the stress-activated map kinase-interacting protein 1 (Sin1) gene expression was markedly decreased. In the EW, EW + EtOH, and chronic ethanol groups, there was a profound increase in the protein expression of mTOR, Sin1, PKCε, and phosphorylated PKCε (Ser729). The PKCε gene and protein expressions showed a statistically significant moderate association, according to a correlation analysis. Our results suggest that an elevated PKCε protein expression in the amygdala during EW and EW + EtOH occurred at the transcriptional level. However, an elevation in the PKCε protein expression, but not its mRNA, after chronic ethanol intake warrants further investigation to fully understand the signaling pathways during different episodes of AUD.

## 1. Introduction

Characteristics of alcohol use disorder (AUD) include a loss of control over binge ethanol consumption, resulting in various social and health harms [1]. In 2017, an estimated 107 million people worldwide suffered from AUD, which was attributed to 2.84 million deaths globally [2]. Alcohol consumption is projected to further increase in 2030 in the Western Pacific and Southeast Asian regions, posing a greater threat to global healthcare [3,4]. Similar to many psychiatric disorders [5,6], over time, a large number of studies have been conducted to elucidate the mechanism of AUD, involving many neurochemical changes [7,8]. Nevertheless, many aspects of ethanol dependence continue to perplex the scientific and healthcare communities [9].

PKC epsilon (PKCε) is abundantly present in the brain regions associated with addiction and drug self-intake, such as the hippocampus, nucleus accumbens, frontal cortex, and amygdala [10,11]. Several studies have reported that PKCε mediates binge ethanol intake and ethanol-induced neuroadaptations [12,13]. Previously, we reported elevated total and phosphorylated PKCε (Ser729) during ethanol withdrawal (EW)-induced anxiety in rats’ amygdala [11]. The PKCε gene was inversely associated with a low response to alcohol, hence indicating its role in the development of tolerance [14]. Ethanol directly alters the subcellular translocation of PKCε [11] and the ensuing cellular activities, thus causing ethanol-induced neuroplasticity [12,15].

Prior to its subcellular translocation, PKCε has to be phosphorylated at Thr566 in the activation loop (AL), Thr710 in the turn motif (TM), and Ser729 in the hydrophobic motif (HM), followed by diacylglycerol (DAG)-induced activation [16]. Existing research indicates that PKCε is first phosphorylated by 3′-phosphoinositide-dependent kinase 1 (PDK1) at AL [17,18], followed by subsequent autophosphorylation at TM and HM (Ser729; the last point of phosphorylation prior to activation) [17]. The administration of Ly294002 (inhibitor of PDK1) and depletion of PDK1 by SiRNA significantly reduced basal levels of novel protein kinase C (PKC) in breast cancer cells [18]. A mammalian target of rapamycin complex 2 (mTORC2) was reported to directly act as an upstream kinase in the phosphorylation of PKCε at TM and HM [19]. mTORC2 consists of stress-activated protein kinase interacting protein (Sin1), mammalian lethal with Sec13 protein 8 (mLST8), mammalian target of rapamycin (mTOR), rapamycin-insensitive companion of mTOR (Rictor), DEP-domain-containing mTOR interacting protein (Deptor), and protein observed with Rictor-1 (protor-1) [19].

In fact, mTOR and Sin1 are the core proteins facilitating TM and HM phosphorylation of PKCε [20,21]. Ikenoue et al. (2008) [21] first reported that the Sin1 and Rictor subunits of mTORC2 are needed for the regulation of protein expression of PKCε and its phosphorylation. In line with this finding, Cameron et al. [22] reported that Sin1 directly interacts with the kinase domain of PKCε via a conserved region in the middle (CRIM). Neither the *C*-terminal PH domain nor the *N*-terminal mTORC binding domain interact robustly with PKCε [22]. The CRIM region is responsible for the selection and recruitment of mTORC2 substrates [22,23]. Disruption of the Sin1 interaction with PKC-inhibited phosphorylation of PKC by mTORC2 occurs despite the retention of Rictor in that complex [22]. In response to ethanol, the expression of vital subunits of mTORC2, including mTOR and Sin1, was shown to be upregulated in C2C12 mouse myoblasts [24]. Another study found that the microinfusion of the mTORC2 activator (A-445634) in the dorsal medial striatum (DMS) boosted ethanol consumption, while the bilateral genetic deletion of mTORC2 in the DMS decreased ethanol intake without influencing water or saccharin intake, suggesting that mTORC2 regulates binge ethanol intake without affecting the pleasure sensations caused by natural reinforcers [25]. To date, no study has reported the effects of ethanol exposure on changes in the expression of mTORC2, especially during various stages of alcohol consumption. Most recent findings have focused on exploring the role of mTORC1 in alcohol dependence [26,27]

In order to fill such gaps, we aimed to understand the ethanol-induced changes in protein and gene expressions of PKCε and mTORC2 in the amygdala during various stages of alcohol addiction.

## 2. Materials and Methods

### 2.1. Materials

The kits for the qPCR analysis and pre-designed primers were obtained from Biosystems (Cumming, GA, USA) and Sigma-Aldrich (St. Louis, MO, USA), respectively. The antibodies (rabbit monoclonal antibody to PKCε, rabbit polyclonal antibody to mTOR, rabbit polyclonal antibody to Sin1, rabbit polyclonal antibody to beta-actin, and goat polyclonal antibody to rabbit IgG) used in the western blot analysis were purchased from Abcam, UK, and the rabbit polyclonal antibody to phosphorylated PKCε Ser729 was obtained from Thermo Scientific (Waltham, MA, USA).

### 2.2. Animal Preparation

Seven-week-old male *Wistar* rats (200–300 g) were obtained from the Laboratory Animal Research Unit, Universiti Kebangsaan Malaysia (UKM). The animals were housed individually at a constant room temperature of 24 °C and kept in a 12 h light–dark cycle with the source of light on between 1900 and 0700. Prior to the experiment, the animals had at least seven days to adapt to the environment. The UKM Animal Ethics Committee gave its approval for all animal techniques used in this study. (FISIO/PP/2016/JAYAKUMAR/28-SEPT./777-NOV.-2016-JULY-2018).

### 2.3. Preliminary Study

In order to establish the peak withdrawal phase prior to the molecular analysis, three groups of animals (*n* = 8 per group) were observed hourly for 12 h to look for withdrawal symptom presentations. Group 1: Rats received modified liquid diet (MLD) that did not contain ethanol. Group 2: MLD-containing ethanol was administered to the animals. Group 3: One hour before the peak withdrawal period, rats received MLD with ethanol and were administered 2.5 g/kg (20% *v*/*v*) of ethanol. After 20 days of continuous ethanol treatment, the rats in Groups 2 and 3 received isocaloric carbohydrate on day 28 in place of the ethanol. The standard MLD was administered to Group 1 rats without ethanol. Following the withdrawal, the rats were monitored every hour for 12 h. During each observation session, the rats were simultaneously assessed for the wide-ranging behavioral symptoms of abnormal posture, abnormal gait, tremors, agitation, stereotyped behavior, and stiffness of the tail. The EW-induced behavioral changes were evaluated based on a scoring system (Supplementary Table S1), and the scoring was carried out by an experienced observer blinded to the experimental conditions [28,29,30].

### 2.4. Experimental Groups

Animals were allocated into five different groups (*n* = 6 per group). The control and acute ethanol groups were given MLD without ethanol for 27 days. On day 28, the control rats were administered with normal saline, whereas the remaining rats assigned to the acute ethanol group were given ethanol (2.5 g/kg, 20% *v*/*v* in saline) via the intraperitoneal route at the 6th hour (following the EW group). In the chronic ethanol group, the rats were fed with control MLD for seven days, and ethanol was gradually introduced to the MLD from 2.4% (days 8–10) to 4.8% (days 11–13) and to 7.2% (days 14–27). On day 28, an acute ethanol (2.5 g/kg, 20% *v*/*v* in saline) injection was given (at 0 h of EW) to ensure the continuous presence of ethanol. The rats assigned to ethanol withdrawal (EW) and ethanol withdrawal with acute ethanol (EW + EtOH) groups were fed with MLD as described for the chronic group. However, on day 28, ethanol was withdrawn from the MLD (hence, 0 h into EW) and replaced with isocaloric MLD consisting of maltodextrin and sucrose. At the 6th hour from the last ethanol intake, the EW group was administered with normal saline, whereas the EW + EtOH group was administered with acute ethanol (2.5 g/kg, 20% *v*/*v* in saline). At the 7th hour into EW, all the animals were administered sodium pentobarbital (100 mg/kg; intraperitoneal), followed by the collection of serum through retro-orbital blood to assess serum ethanol levels using the EnzyChrom ethanol assay kit (ECET-100) obtained from BioAssay (Hayward, CA, USA). Every day in the morning, the rats’ body weight was measured. Their daily ethanol intake was expressed in grams per kilogram per day. The administration of MLD and the dosages and times of intraperitoneal injections were based on [11,31]. Amygdalae were collected bilaterally, as described in [11], following decapitation. The administration of MLD and the time of drug administration are shown in Table 1.

### 2.5. Modified Liquid Diet (MLD)

The ethanol-containing MLD comprised low-fat Dutch lady cow’s milk powder, maltodextrin, sucrose, and ethanol 95.6%, based on our previously published works [11,31]. The total caloric content of the mixture is about 1070 kCal/L (for MLD without ethanol: milk powder—54.95%, carbohydrate—45.04%; for MLD containing EtOH milk powder, D8-10: milk powder—54.95%, ethanol—12.02%, carbohydrate—33.02%; D11-D13: milk powder—54.95%, ethanol—24.04%, carbohydrate—21%; D14–D27: milk powder—54.95%, ethanol—36.1%, carbohydrate—8.98%). The MLD was a modification of the ethanol-containing liquid diet proposed by Uzbay and Kayaalp [32].

### 2.6. RNA Extraction and qPCR Analysis

After dissection, the amygdalae were immediately submerged in RNA stabilization reagent (RNAlater) (10 µL per 1 mg of tissue) and incubated overnight at 2–8 °C. The tissue was removed from the reagent and transferred into a 1.5 mL tube. RNA extraction was performed using a Nucleospin^®^ RNA kit, and cDNA synthesis was performed using a qPCR BIO cDNA synthesis kit according to the manufacturer’s guidelines. The qPCR steps were carried out using qPCRBIO SyGreen Blue Mix separate-ROX (Bio-Rad CFX Manager 3.1). The experiment was designed according to 96-well formats. The target genes (PKCε, mTOR, and Sin1) were run together with a reference gene (beta-actin) (Supplementary Table S2). The average of the relative gene expression was measured using the 2^−ΔΔCq^ (Livak) method [33].

### 2.7. Western Blot Analysis

The protein was extracted from the amygdalae using radioimmunoprecipitation assay (RIPA) buffer 1X, and the concentration of the protein was determined using Bradford reagent. A 30 µg aliquot of the homogenized sample of each target protein was electrophoresed at 100 Volts for 15 min and immediately changed to 140 Volts for about 30 min using the Bio-Rad Power Pac Basic model. The electrophoretic transfer was conducted at 100 Volts for 90 min. Then, the PVDF membranes for mTOR, PKCε, phosphorylated PKCε Ser729, and Sin1 were incubated with rabbit polyclonal antibody to mTOR (1/6000 in 3% BSA/TBST), rabbit monoclonal antibody to PKCε (1/7000 in 3% BSA/TBST), rabbit polyclonal antibody to phosphorylated PKCε Ser729 (1/1000 in 3% BSA/TBST), and rabbit polyclonal antibody to Sin1 (1/5000 in 3% BSA/TBST), respectively, overnight at 4 °C. Following this, the membranes were incubated with a secondary antibody, goat anti-rabbit IgG H&L (Alexa Flour^®^ 568) (1/500,000 in 3% BSA/TBST), for one hour at room temperature prior to chemiluminescent imaging. After capturing the image, the membranes were washed and incubated with rabbit polyclonal antibody to beta-actin (1/5000 in 3% BSA/TBST) for two hours at room temperature. Then, the membranes were incubated again with goat anti-rabbit IgG H&L (Alexa Flour^®^ 568) (1/500,000 in 3% BSA/TBST), and we proceeded with chemiluminescent imaging. The integrated density values (IDV) of the PKCε, phosphorylated PKCε (Ser729), mTOR, Sin1, and beta-actin (β-Actin) proteins were measured using the Amersham Imager 600 software. The expression of the protein of interest was normalized with β-Actin. The expression of phosphorylated protein was divided by the expression of total protein to obtain the ratio. All final values were obtained relative to the untreated group (control).

### 2.8. Statistical Analysis

The results are presented as mean ± SEM. *p* < 0.05 was considered to be statistically significant. All data were tested for normality using Statistical Package for the Social Sciences (SPSS) version 27. Data for the changes in body weight were analyzed using a paired *t*-test. The data for daily MLD intake, daily ethanol intake, serum ethanol level, qPCR, and Western blot were analyzed using a one-way analysis of variance (ANOVA) and post-hoc Tukey’s test. The relationship between gene and protein expressions was analyzed using Pearson’s correlation.

## 3. Results

### 3.1. Assessment of EW Symptoms

**Figure 1:**
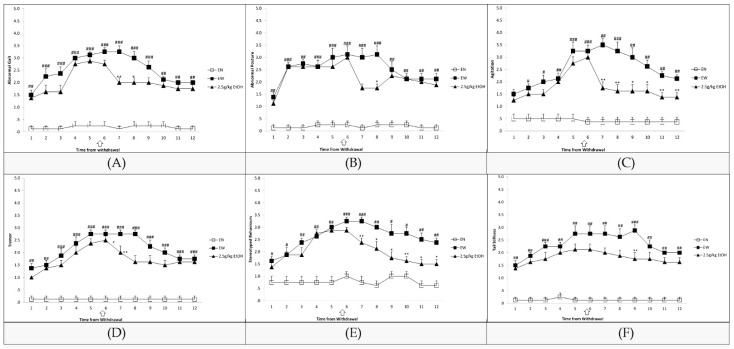
(**A**) Score for abnormal gait during ethanol withdrawal. (**B**) Score for abnormal posture during ethanol withdrawal. (**C**) Score for agitation during ethanol withdrawal. (**D**) Score for tremor during ethanol withdrawal. Drugs were administered at sixth hour from withdrawal (indicated by an arrow). (**E**) Score for stereotyped behaviors during ethanol withdrawal. (**F**) Score for tail stiffness during ethanol withdrawal. Values are represented by mean ± SEM. (EN = rats given non-ethanol containing MLD and administered with saline; EW = ethanol withdrawal (ethanol withdrawn rats administered with normal saline); 2.5 g/kg EtOH = ethanol withdrawn group treated with 2.5 g/kg ethanol; * *p* < 0.05, ** *p* < 0.01 vs. EW; # *p* < 0.05, ## *p* < 0.01, ### *p* < 0.001 vs. EN, Kruskal–Wallis one-way ANOVA and Mann–Whitney U Test).

The severity of withdrawal symptoms increased from the first to the seventh hour after the last ethanol intake and then decreased from the eighth to the twelfth hour (Figure 1A–F). When compared to the ethanol-naive group, ethanol withdrawal significantly increased the scores of abnormal gait (1–12 h), abnormal posture (1–12 h), agitation (2–12 h), tremor (1–12 h), stereotyped behaviors (1–12 h), and tail stiffness (1–12 h). The arrow sign in the data indicates that 2.5 g/kg ethanol (ip) was administered at hour six, which led to a considerable decline in the scores for abnormal posture (8 h), tremors (7, 8 h), agitation (7–12 h), stereotyped behaviors (7–12 h), tail stiffness (9 h), and abnormal gait (7 h, 8 h).

### 3.2. Body Weight Changes

The mean body weight changes in ethanol-fed and ethanol naive animals over the period between days 1 and 27 of MLD intake (equivalent to D1 and D20 of exposure to an ethanol-containing diet) are shown in Table 2. The ethanol-naive (control and acute alcohol) groups (*p* > 0.05) weighed 0.6% higher than their initial weights at the end of the study, whereas the ethanol-fed (chronic, EW, and EW + EtOH) animals (*p* < 0.01) weighed significantly (11.98%) less than their initial body weights. In our previous studies, we noticed a similar trend where ethanol-fed animals weighed less than their initial weights compared to ethanol-naive animals [11,31].

### 3.3. Daily MLD Intake

Table 3 shows a significant difference in MLD intake across the treatment groups (F (4;25) = 15.0951; *p* < 0.001). The chronic ethanol group consumed significantly less MLD compared to the acute ethanol group (*p* < 0.05), but not the control group. The daily MLD intake by EW and EW + EtOH groups was profoundly less compared to the control (*p* < 0.01) and acute ethanol (*p* < 0.01) groups. No significant difference was noticed in MLD intake between the control and acute ethanol groups (*p* > 0.05)

### 3.4. Daily Intake of Ethanol

There was no profound difference in the average ethanol consumed across the groups (F (2,15) = 1.827; *p* > 0.05) (see Table 4).

### 3.5. Serum Ethanol Concentration

There was a significant difference in the serum ethanol concentration across the groups (F (3,20) = 40.7869, *p* < 0.01) (see Table 5). The highest value was seen in the chronic group (*p* < 0.01) compared to the acute ethanol, EW, and EW + EtOH groups. The serum ethanol concentration of chronic ethanol (*p* < 0.01) and EW + EtOH (*p* < 0.05) groups was significantly higher than that of the acute ethanol group.

### 3.6. Mean Relative mRNA Level in the Rats’ Amygdalae

**Figure 2:**
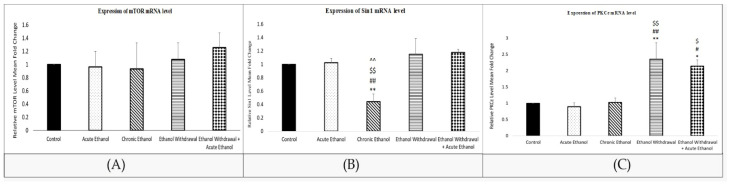
(**A**) Relative mTOR mRNA levels in the amygdalae of rats. Each bar indicates mean ± SEM (control group: control rats fed with MLD without ethanol and treated with saline; acute ethanol: rats fed with MLD without ethanol and treated with acute ethanol (2.5 g/kg ethanol, 20% *v*/*v*); chronic ethanol = rats fed with ethanol containing MLD for 20 days; EW = rats fed with ethanol containing MLD for 20 days and withdrawn from ethanol on day 21; EW + EtOH = rats fed with ethanol containing MLD for 20 days, withdrawn from ethanol on day 21, and administered with acute ethanol (2.5 g/kg, 20% *v*/*v*)). One-way ANOVA and post hoc Tukey’s test: relative mTOR mRNA levels (F (4,25) = 0.252, *p* > 0.05 compared to control group). (**B**) Relative Sin1 mRNA levels in the amygdalae of rats. Each bar indicates mean ± SEM (control group: control rats fed with MLD without ethanol and treated with saline; acute ethanol: rats fed with MLD without ethanol and treated with acute ethanol (2.5 g/kg ethanol, 20% *v*/*v*); chronic ethanol = rats fed with ethanol containing MLD for 20 days; EW = rats fed with ethanol containing MLD for 20 days and withdrawn from ethanol on day 21; EW + EtOH = rats fed with ethanol containing MLD for 20 days, withdrawn from ethanol on day 21, and administered with acute ethanol (2.5 g/kg, 20% *v*/*v*)). One-way ANOVA and post hoc Tukey’s test: relative Sin1 mRNA levels (F (4,25) =10.7393, ** *p* < 0.01 compared to control group; ## *p* < 0.01 compared to acute group; $$ *p* < 0.01 compared to EW group; and ^^ *p* < 0.01 compared to EW + EtOH). (**C**) Relative PKCε mRNA level in the amygdalae of rats. Each bar indicates mean ± SEM (control group: control rats fed with MLD without ethanol and treated with saline; acute ethanol: rats fed with MLD without ethanol and treated with acute ethanol (2.5 g/kg ethanol, 20% *v*/*v*); chronic ethanol = rats fed with ethanol containing MLD for 20 days; EW = rats fed with ethanol containing MLD for 20 days and withdrawn from ethanol on day 21; EW + EtOH = rats fed with ethanol containing MLD for 20 days, withdrawn from ethanol on day 21, and administered with acute ethanol (2.5 g/kg, 20% *v*/*v*)). One-way ANOVA and post hoc Tukey’s test: relative PKCε mRNA level (F (4,25) = 7.597; * *p* < 0.05, ** *p* < 0.01 compared to control group; # *p* < 0.05, ## *p* < 0.01 compared to acute ethanol group; $ *p* < 0.05, $$ *p* < 0.01 compared to chronic ethanol group).

As shown in Figure 2A, there was no profound difference in relative mTOR gene expression (F (4,25) = 0.252, *p* > 0.05). However, there were significant differences in PKCε (F (4,25) = 7.597, *p* < 0.01) and Sin1 (F (4,25) = 10.7393, *p* < 0.01) gene expression across the groups. As for Sin1, significantly less expression was found in the chronic ethanol group compared to the control (*p* < 0.01), acute ethanol (*p* < 0.01), EW (*p* < 0.01), and EW + EtOH (*p* < 0.01) groups (Figure 2B). A significant upregulation in PKCε mRNA levels was recorded in the EW (*p* < 0.01) and EW + EtOH (*p* < 0.05) groups compared to the control, acute ethanol, and chronic ethanol groups. There was no significant difference between the control, acute ethanol, and chronic ethanol groups in the gene expression of PKCε (*p* > 0.05) (Figure 2C).

### 3.7. Mean Relative Protein Level in the Rats’ Amygdalae

**Figure 3:**
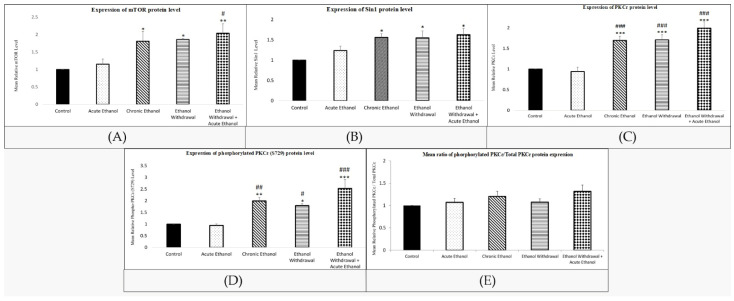
(**A**) The changes in protein expression of mTOR in whole cell lysate of amygdalae. Each bar indicates mean ± SEM (control group: rats fed with MLD with-out ethanol and treated with saline; acute ethanol: rats fed with MLD without ethanol and treated with acute ethanol (2.5 g/kg ethanol, 20% *v*/*v*); chronic ethanol = rats fed with ethanol containing MLD for 20 days; EW = rats fed with ethanol containing MLD for 20 days and withdrawn from ethanol on day 21; EW + EtOH = rats fed with ethanol containing MLD for 20 days, withdrawn from ethanol on day 21, and administered with acute ethanol (2.5 g/kg, 20% *v*/*v*)); one-way ANOVA and post-hoc Tukey’s test: mTOR (F (4,25) = 6.027; * *p* < 0.05, ** *p* < 0.01 compared to control group; # *p* < 0.05 compared to acute ethanol group). (**B**) The changes in protein expression of Sin1 in whole cell lysate of amygdalae. Each bar indicates mean ± SEM (control group: rats fed with MLD with-out ethanol and treated with saline; acute ethanol: rats fed with MLD without ethanol and treated with acute ethanol (2.5 g/kg ethanol, 20% *v*/*v*); chronic ethanol = rats fed with ethanol containing MLD for 20 days; EW = rats fed with ethanol containing MLD for 20 days and withdrawn from ethanol on day 21; EW + EtOH = rats fed with ethanol containing MLD for 20 days, withdrawn from ethanol on day 21, and administered with acute ethanol (2.5 g/kg, 20% *v*/*v*)); one-way ANOVA and post-hoc Tukey’s test: Sin1 level (F (4,25) = 4.840; * *p* < 0.05 compared to control group). (**C**) The changes in protein expression of PKCε in whole cell lysate of amygdalae. Each bar indicates mean ± SEM (control group: rats fed with MLD with-out ethanol and treated with saline; acute ethanol: rats fed with MLD without ethanol and treated with acute ethanol (2.5 g/kg ethanol, 20% *v*/*v*); chronic ethanol = rats fed with ethanol containing MLD for 20 days; EW = rats fed with ethanol containing MLD for 20 days and withdrawn from ethanol on day 21; EW + EtOH = rats fed with ethanol containing MLD for 20 days, withdrawn from ethanol on day 21, and administered with acute ethanol (2.5 g/kg, 20% *v*/*v*)); one-way ANOVA and post-hoc Tukey’s test: PKCε level (F (4,25) = 17.997; *** *p* < 0.001 compared to control group; ### *p* < 0.001 compared to acute ethanol group). (**D**) The changes in protein expression of phosphorylated PKCε in whole cell lysate of amygdalae. Each bar indicates mean ± SEM (control group: rats fed with MLD with-out ethanol and treated with saline; acute ethanol: rats fed with MLD without ethanol and treated with acute ethanol (2.5 g/kg ethanol, 20% *v*/*v*); chronic ethanol = rats fed with ethanol containing MLD for 20 days; EW = rats fed with ethanol containing MLD for 20 days and withdrawn from ethanol on day 21; EW + EtOH = rats fed with ethanol containing MLD for 20 days, withdrawn from ethanol on day 21, and administered with acute ethanol (2.5 g/kg, 20% *v*/*v*)); one-way ANOVA and post-hoc Tukey’s test: phosphorylated PKCε (Ser729) level (F (4,25) = 13.293; * *p* < 0.05, ** *p* < 0.01, *** *p* < 0.001 compared to control group; # *p* < 0.05, ## *p* < 0.01, ### *p* < 0.001 compared to acute ethanol group). (**E**) Mean ratio of phosphorylated PKCε/Total PKCε protein expression in whole cell lysate of amygdalae. Each bar indicates mean ± SEM (control group: rats fed with MLD with-out ethanol and treated with saline; acute ethanol: rats fed with MLD without ethanol and treated with acute ethanol (2.5 g/kg ethanol, 20% *v*/*v*); chronic ethanol = rats fed with ethanol containing MLD for 20 days; EW = rats fed with ethanol containing MLD for 20 days and withdrawn from ethanol on day 21; EW + EtOH = rats fed with ethanol containing MLD for 20 days, withdrawn from ethanol on day 21, and administered with acute ethanol (2.5 g/kg, 20% *v*/*v*)); one-way ANOVA and post-hoc Tukey’s test: Phosphorylated PKCε (Ser729)/total PKCε expression ratio (F (4,25) = 1.133; *p* > 0.05 compared to control group.

Across the groups, there was a significant difference in mean relative protein expression of mTOR (F (4,25) = 6.027, *p* < 0.05), PKCε (F (4,25) = 17.997; *p* < 0.001), phosphorylated PKCε (Ser729) (F (4,25) = 13.293; *p* < 0.001), and Sin1 (F (4,25) = 4.840; *p* < 0.05), except for the phosphorylated/PKCε ratio (F (4,25) = 1.133; *p* > 0.05). mTOR expression was particularly elevated in the chronic ethanol (*p* < 0.05), EW (*p* < 0.05), and EW + EtOH (*p* < 0.01) groups compared to the control (Figure 3A). A profound increase in Sin1 protein expression was noticed in the chronic ethanol (*p* < 0.05), EW (*p* < 0.05), and EW + EtOH (*p* < 0.05) groups compared to the control group (Figure 3B). The expression of PKCε was significantly elevated in the chronic ethanol (*p* < 0.001), EW (*p* < 0.001), and EW + EtOH (*p* < 0.001) groups compared to the control and acute ethanol groups (Figure 3C). Similarly, an upregulation of phosphorylated PKCε (Ser729) was observed in the chronic ethanol (*p* < 0.01), EW (*p* < 0.05), and EW + EtOH (*p* < 0.001) groups compared to the control and acute ethanol groups (Figure 3D). However, there was no significant difference in the phosphorylated PKCε (Ser729)/total PKCε expression ratio (F (4,25) = 1.133; *p* > 0.05) across the groups (Figure 3E).

### 3.8. Correlation Analysis between Gene and Protein Expressions

The Pearson’s correlation analysis revealed a weak correlation between mTOR protein and gene expressions, which was statistically insignificant (r = 0.227, *n* = 30, *p* > 0.05). However, for PKCε, there was a statistically significant moderate correlation between its gene and protein expressions (r = 0.472, *n* = 30, *p* < 0.01). For Sin1 gene and protein expressions, there was a weak correlation without any statistical significance (r = 0.183, *n* = 30, *p* > 0.05).

## 4. Discussion

In the current study, the serum ethanol levels of the chronic ethanol group were within a similar range of values to those previously reported by [34], where rats fed with ethanol-containing diet for four weeks followed by three binge intragastric administrations (12 h intervals between each injection) of ethanol (5 g/kg) recorded 540 mg/dl. In the present study, serum ethanol levels were slightly higher after 20 days of chronic ethanol consumption and an acute ethanol injection (2.5% *v*/*v*, 20%) via the intraperitoneal route, and the blood sample was collected an hour after the ethanol injection. In the previous study, by Aroor and Shukla [34], ethanol was administered through the oral cavity, and the blood sample was collected 4 h after the last ethanol administration. Therefore, the intraperitoneal route of the last ethanol administration, along with the shorter duration of blood sample collection, could have resulted in the higher serum ethanol levels in the present study. The amount of ethanol consumed did not differ noticeably between the ethanol-containing MLD-fed groups.

In the current study, there was a significant increase in total and phosphorylated PKCε (Ser729) protein and gene expressions in the EW and EW + EtOH groups. A correlation analysis showed a significant moderate association between the gene and protein expressions of PKCε, indicating that the increase took place at the transcriptional level. Transcription of the PKCε gene is regulated by different promoter regions [35] with specific functional sites for the Jun, nuclear factor kappa β (NF-ĸβ), Stat3, Mtf, and Sp1 transcription factors, cAMP-response element-binding (CREB) [36], and activator protein 1 (AP-1). Among the transcription factors, NF-ĸβ, CREB, and AP-1 have been associated with prolonged ethanol consumption-induced gene transcription and reported to be regulated through MAPKs and PI3K pathways [37,38,39]. PKCs activate the ERK pathway indirectly by phosphorylating Raf, which causes the conversion of MAP3K to MAP2K and MAPK (MEK1/MEK2). The activation of MAPK subsequently leads to the phosphorylation of ERK1/2 [40,41]. When ERK1/2 is phosphorylated, it translocates to the nucleus and activates transcription factors including NF-ĸβ, CREB, and AP-1 [42,43]. According to existing research, PKCε activates NF-sĸβ through the ERK1/2 pathway in prostate cancer and rabbit cardiomyocytes [44,45]. Moreover, in cortical neuronal cell cultures, PKCε also stimulates NF-ĸβ via the ERK1/2 cascade [46]. ERK1/2 is abundantly expressed in limbic brain regions such as the nucleus accumbens, prefrontal cortex, and amygdala, which are involved in drug dependence [47,48]. Abstinence from prolonged ethanol intake dramatically has increased ERK phosphorylation levels in the amygdala, cerebellum, and striatum, with the highest activity observed in the amygdala [49,50].

Another important finding from this study is that the chronic intake of ethanol did not alter the gene expression of PKCε but significantly increased the protein expression of PKCε, which suggests an alternate signaling pathway taking place in the amygdala during prolonged alcohol intake and abstinence. In line with this, it was previously reported that acute and chronic ethanol administrations reduce ERK1/2 activity in the amygdala, cerebellum, dorsal striatum, hippocampus, and frontal cortex, which, in turn, downregulates the activities of transcription factors associated with ERK1/2 [49,51,52]. Parallel to this, in our study, the gene expression of PKCε following acute and chronic ethanol intake was almost similar to that of the control group. Therefore, the elevated protein levels of PKCε and phosphorylated PKCε were regulated at the translational level. Ethanol activates PLC, which catalyzes the conversion of PIP_2_ to generate PIP_3_ and DAG [53]. DAG acts as a natural activator of PKCε [54]. Chronic ethanol exposure lowers PIP_2_-specific Phospholipase C (PLC) activity in mouse brains, consequently reducing intracellular levels of DAG as well [55]. Consistent with these findings, baseline DAG levels were demonstrated to be sufficient for PKC activation if levels of PKCs were increased [56]. The dephosphorylation and downregulation of PKCs have been reported in prior work following an increase in DAG levels [57]. The autoinhibitory pseudo substrate is released from the substrate binding cavity by the binding of DAG to PKCs, changing the conformation from closed to open and becoming sensitive to the dephosphorylation and degradation processes [58]. The presence of leucine-rich repeat protein phosphatases (PHLPP) and PH domain dephosphorylate novel PKCs, specifically, at HM, which destabilizes the kinase, results in further dephosphorylation at AL and TM by PP2A-type phosphatases [59] and degradation [59]. In line with this, reduced PHLPP activity was correlated with an increase in the expression of PKCε in normal breast epithelial and colon cancer cells [59]. In line with this, during chronic ethanol consumption, levels of DAG are reduced and PKCε is translocated away from PHLPP sites [60]. Therefore, it is likely that ethanol may “protect” PKCε from degradation to maintain high intracellular PKCε levels and mediate downstream signaling pathways.

In the present study, there were no significant differences across the groups in the ratio of phosphorylated PKCε (Ser729)/total PKCε; however, a significant increase was seen in both total and phosphorylated PKCε (Ser729) in the EW and EW + EtOH groups. These findings suggest that an increase in PKCε phosphorylation took place in the aforementioned groups (since the ratio is still higher than 1); however, the expression of total PKCε also almost equally increased (clearly shown by our protein expression results). Prolonged exposure to ethanol reduces PIP2-specific PLC activity, leading to the reduced synthesis of DAG [55]. DAG is essential for PKCε activation; however, an increase in DAG also accelerates the degradation of PKCε. In line with this, long-term exposure to ethanol decreased the expression of DAG [60]. Based on this study and existing literature, it can be suggested that PLC activity would remain low in the amygdala of rats fed with ethanol for 20 days. Hence, total PKCε was significantly upregulated to provide a sufficient reserve pool of PKCε available to be phosphorylated and activated even in the presence of a baseline level of DAG [11,56].

In the current study, prolonged ethanol exposure may have increased levels of phosphorylated PKCε (at the S729 site) through the activation of upstream kinases, such as PI3K, PDK1, or mTORC2 pathways, providing an alternative explanation for our findings. PKCε must undergo phosphorylation at AL, TM, and HM to become catalytically active and stable [61]. Phosphorylation at AL is catalyzed by PDK1 and followed by subsequent autophosphorylation at TM and HM. PDK1 is activated by PI3K, and PI3K is activated via G-protein coupled receptors and tyrosine kinase receptors [62]. Repeated bouts of ethanol intake increase PI3K activity in the nucleus accumbens [63]. Activation of PI3K induces a series of cellular reactions involving numerous downstream kinases, including PDK1 and mTORC2 [64,65]. The phosphorylation of PKCs at TM and HM via autophosphorylation has raised the question of whether autophosphorylation is taking place at those sites [66]. mTORC2 was shown to phosphorylate PKCε at TM and HM [25]. mTORC2 consists of Rictor and Sin1, and Sin1 is crucial in TM and HM phosphorylation of PKCε [21,25]. In line with this, there was also a significant increase in the protein expression of mTOR and Sin1 in the experimental groups with elevated phosphorylated PKCε in this study (S729). However, in the present study, there was no causal link between mTORC2 and phosphorylation of PKCε (S729).

To date, this is the first study to report the direct effects of ethanol on the protein expression of mTORC2 subunits in brain tissue. Previously, the activities of mTORC2 have been reported to be significantly increased in DMS following repeated bouts of binge ethanol intake and withdrawal in 8–10-week-old male C57BL/6J mice. In this study, ethanol was given to the mice using an IA20%-2BC procedure consisting of 20% ethanol (*v*/*v*) (average consumption of ethanol was 14 g/kg/day) in tap water for 7–8 weeks with intermittent abstinence during the feeding period. The mTORC2 activities were measured through the protein levels of mTORC2′s well-characterized substrates, such as Akt phosphorylated at Serine 473 (Ser473 Akt) [19,67], serum and glucocorticoid-induced protein kinase 1 phosphorylated at the Serine 422 residue (Ser422 SGK1) [68], and, also, the substrate of Akt, the phosphorylated form of Glycogen synthase kinase 3 beta (GSK3β) [25]. The expression of the aforementioned proteins was significantly upregulated in DMS following repeated bouts of binge intake of ethanol and withdrawal [25]. In addition, the systemic administration of ethanol and voluntary excessive consumption of ethanol followed by periods of withdrawal in NAc of rats also resulted in an increase in the expression of Ser473 Akt and GSK3β [69]. In line with these findings, a study by Hong Brown and colleagues (2010) [70] also reported a significant elevation in the expression of mTOR and Sin1 in C2C12 following ethanol exposure (100 mM) for 18–24 h [70]. In the present study, the ethanol-fed rats consumed an average of 11.38 to 12.65 g/kg day^−1^ for 20 days and achieved 340.13 to 582.69 mg/dl serum ethanol levels.

mTOR is a functional subunit of mTORC1 and mTORC2. Therefore, an increase in the expression of mTOR could also be indicative of an increase in the activities of mTORC1 in the chronic ethanol, EW, and EW + EtOH groups. In the current study, the protein expression of Sin1 was recorded within the range of 1.56–1.63 ± 0.23–0.38, whereas the expression of mTOR was within the range of 1.81–2.04 ± 0.69–0.66, which shows that the expression of mTOR was relatively higher than that of Sin1 for all three experimental groups. In agreement with our findings, binge ethanol consumption was shown to promote mTORC1 activities in the nucleus accumbens [71], not only during the first binge-drinking episode but also during voluntary ethanol ingestion and 24 h after abstinence [72,73]. Furthermore, activities of mTORC1 were also increased in brain regions such as the central amygdala, orbitofrontal cortex, and prelimbic area during the reconsolidation of ethanol-associated memories [74,75]. Existing evidence has indicated the importance of mTORC1 in the regulation of ethanol-induced neuroplasticity, and consequently, the behavioral changes in AUD, and this investigation adds further credence to this by reporting an increase in the expression of mTOR in the amygdala at later stages of AUD.

Current findings indicate that there was a consistent increase in the expression of both the mTOR gene and protein in the order of chronic < EW < EW + EtOH groups. However, only the gene expression of EW and EW + EtOH was higher than the control (statistically insignificant), and the gene expression of chronic was less than the control. Based on this and an insignificant correlation analysis, it can be deduced that an increase in the expression of mTOR took place at the translational stage. For Sin1, the gene expression of chronic ethanol, EW, and EW + EtOH groups was less than that of the control, especially the chronic group, which recorded a significant downregulation in expression; however, its protein expression was markedly increased. Although there is no definitive explanation for this at present, the observed changes in PKCε expression suggest that signaling pathways may differ between chronic alcohol consumption and abstinence conditions. Thus, future studies focused on the pharmacological modulation of PKCε and mTORC2 should carefully consider the potential influence of alcohol consumption and abstinence on these pathways to ensure an accurate interpretation of the results.

## 5. Conclusions

An increase in the phosphorylation of PKCε (Ser729) during the late stages of AUD could be due to the elevated transcription of PKCε, which is most likely mediated by ERK1/2. Contrary to this, a significant increase in PKCε protein expression, but not its gene expression, during chronic ethanol exposure was noticed in the present study. There were also opposing changes in the gene and protein expressions of Sin1 upon chronic alcohol consumption. These findings suggest possible alternative signaling pathways taking place at the cellular level during chronic and withdrawal states of AUD in the amygdala. At present, the increase in ERK1/2 activity in the amygdala during EW can be theoretically related to mediating an increase in the transcription of PKCε and, eventually, the total protein expression of PKCε. Nevertheless, further research is warranted to confirm this in the future.

## Figures and Tables

**Table 1:** Schedule of ethanol and non-ethanol-containing modified liquid diet (MLD) administration based on animal grouping and intraperitoneal injections of saline or acute ethanol (acute EtOH) (2.5 g/kg, 20% *v*/*v*); chronic ethanol (chronic EtOH); EW (ethanol withdrawal); EW + EtOH (ethanol withdrawal group given acute ethanol challenge).

Group	D1–D7	D8–D27	D28		
			0 h	6 h	7 h
Control	MLD	MLD	MLD	Saline	Euthanasia
Acute EtOH	MLD	MLD	MLD	Acute EtOH	Euthanasia
Chronic EtOH	MLD	MLD + EtOH	Acute EtOH	MLD + EtOH	Euthanasia
EW	MLD	MLD + EtOH	MLD	Saline	Euthanasia
EW + EtOH	MLD	MLD + EtOH	MLD	Acute EtOH	Euthanasia

**Table 2:** Body-weight changes.

Group	Day 1 (g)	Day 27 (g)	Body Weight Changes (%)
Ethanol naive (*n* = 12)	266.43 ± 14.92	267.99 ± 9.57	+0.6
Ethanol fed (*n* = 18)	262.53 ± 7.87	231.09 ± 6.16	−11.98 **

** *p* < 0.01, paired *t*-test; ethanol naive: rats fed with MLD without ethanol throughout the MLD intake; ethanol-fed: rats fed with MLD containing ethanol.

**Table 3:** Mean MLD intake from Day 1 to Day 27.

Group	Daily MLD Intake (mL/Day)
Control	67.43 ± 3.23
Acute EtOH	71.06 ± 2.82
Chronic EtOH	59.69 ± 0.48 #
EW	51.04 ± 1.07 **, ##
EW + EtOH	52.04 ± 2.62 **, ##

Mean ± SEM of the volume per day of MLD consumed from day 1 to day 27 of MLD intake. One-way ANOVA, (F (4,25) = 15.0951, *p* < 0.01; ** *p* < 0.01 when compared against control group; # *p* < 0.05 and ## *p* < 0.01 when compared against acute ethanol group) and post-hoc Tukey’s test.

**Table 4:** Mean ethanol intake from Day 8 to Day 27.

Group	Daily Ethanol Intake (g/kg Day^−1^)
Chronic EtOH	12.65 ± 0.44855
EW	12.02 ± 0.26170
EW + EtOH	11.38 ± 0.62499

Mean ± SEM of amount of ethanol consumed per day from day 8 to day 27 of MLD intake. One-way ANOVA (F (2,15) = 1.827; *p* > 0.05), average daily ethanol consumed for 20 days.

**Table 5:** Mean serum ethanol level.

Group	Serum Ethanol Level (mg/dL)
Acute EtOH	285.26 ± 42.10 ##
Chronic EtOH	582.69 ± 70.42
EW	340.13 ± 20.55 ##
EW + EtOH	384.63 ± 29.78 *, ##

Mean ± SEM of serum ethanol level. Serum was collected on day 28 of MLD intake. One-way ANOVA, (F (3,20) = 40.7869; * *p* < 0.05 when compared against acute ethanol group and ## *p* < 0.01 when compared against chronic ethanol group) and post-hoc Tukey’s test.

## Data Availability

Not applicable.

## Supplementary Materials

The following supporting information can be downloaded at https://www.mdpi.com/article/10.3390/nu15133036/s1: Figure S1: A western blot for whole cell lysates of protein expressions of (A) mTOR (B) sin1 (C) PKCε (D) phosphorylated PKCε (ser729) in the rat’s amygdala. The lower panel shows the loading control (β Actin). Figure S2: Score for abnormal gait during ethanol withdrawal. Figure S3: Score for abnormal posture during ethanol withdrawal. Figure S4: Score for agitation during ethanol withdrawal. Figure S5: Score for tremor during ethanol withdrawal. Figure S6: Score for stereotyped behaviours during ethanol withdrawal. Figure S7: Score for tail stiffness during ethanol withdrawal. Figure S8: Relative mTOR mRNA level in the amygdala of rats. Figure S9: Relative Sin1 mRNA level in the amygdala of rats. Figure S10. Relative PKCε mRNA level in the amygdala of rats. Figure S11: The changes in protein expression of mTOR in whole cell lysate of amygdala. Figure S12: The changes in protein expression of Sin1 in whole cell lysate of amygdala. Figure S13: The changes in protein expression of PKCε in whole cell lysate of amygdala. Figure S14: The changes in protein expression of phosphorylated PKCε in whole cell lysate of amygdala. Figure S15: Mean ratio of phosphorylated PKCε/Total PKCε protein expression in whole cell lysate of amygdala. Table S1: Evaluation of EW symptoms. Table S2: Characteristics of the pre-designed gene specific primer used in the present study.
