# Supplementary material for: Alcohol Dependence Modulates Amygdalar mTORC2 and PKCε Expression in a Rodent Model
Source: Nutrients. 2023 Jul 5;15(13):3036. doi: 10.3390/nu15133036 (PMC10346598; doi:10.3390/nu15133036)
Supplement: Supplementary file 1 [file nutrients-15-03036-s001.zip › nutrients-2455939-supplementary Figures S2-S15.pdf]

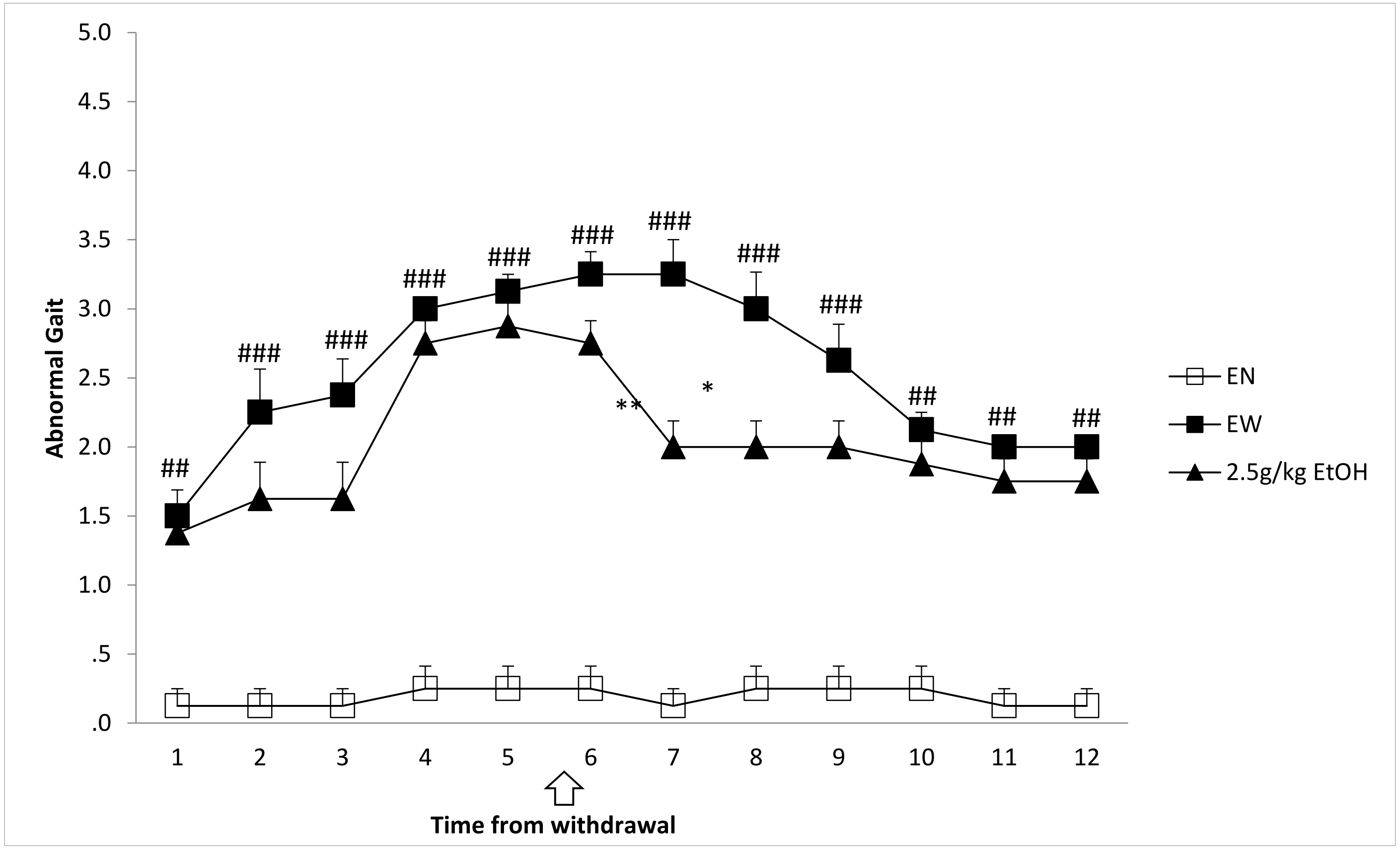

Supplementary Figure S2. Score for abnormal gait during ethanol withdrawal.

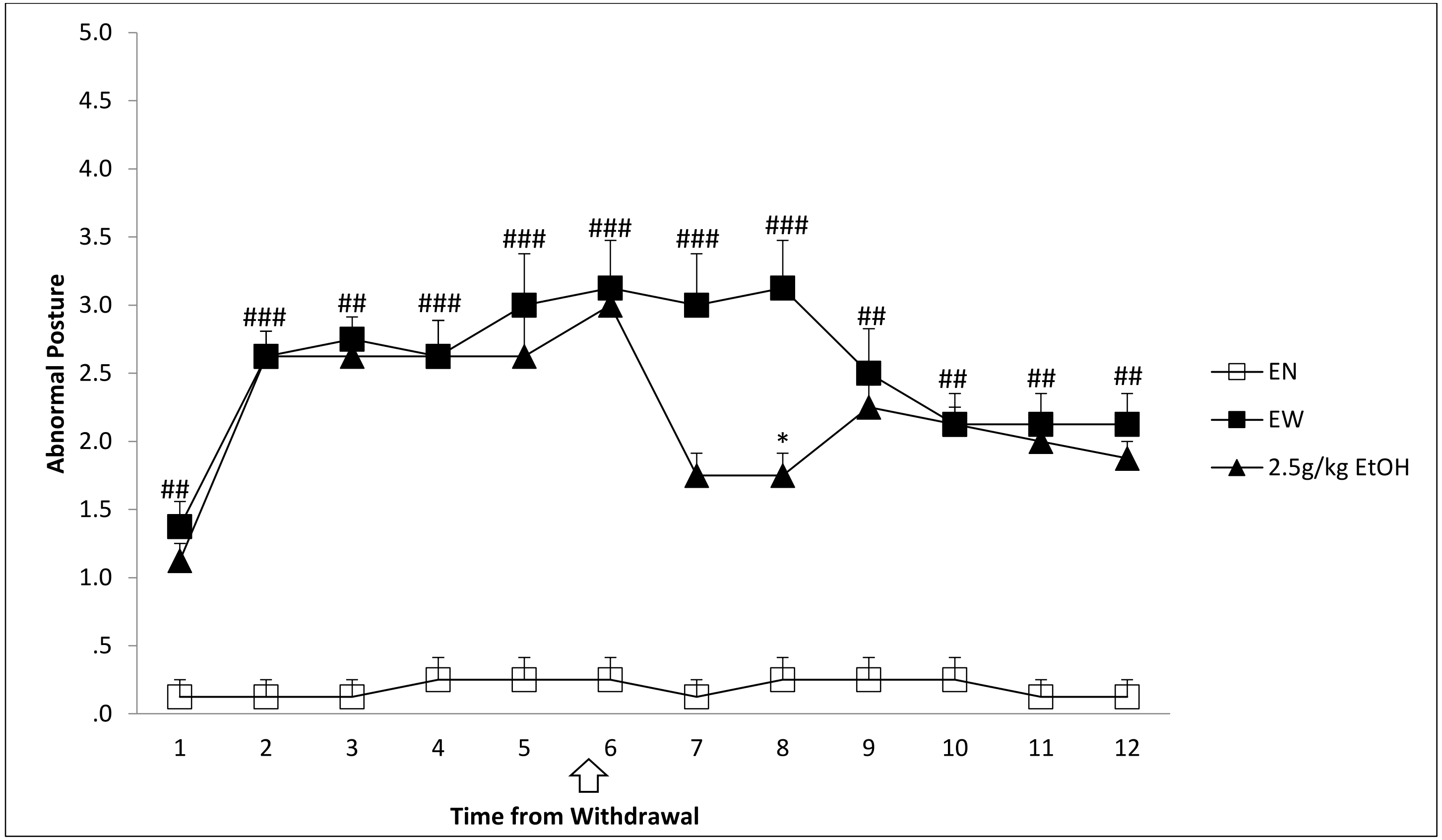

Supplementary Figure S3. Score for abnormal posture during ethanol withdrawal.

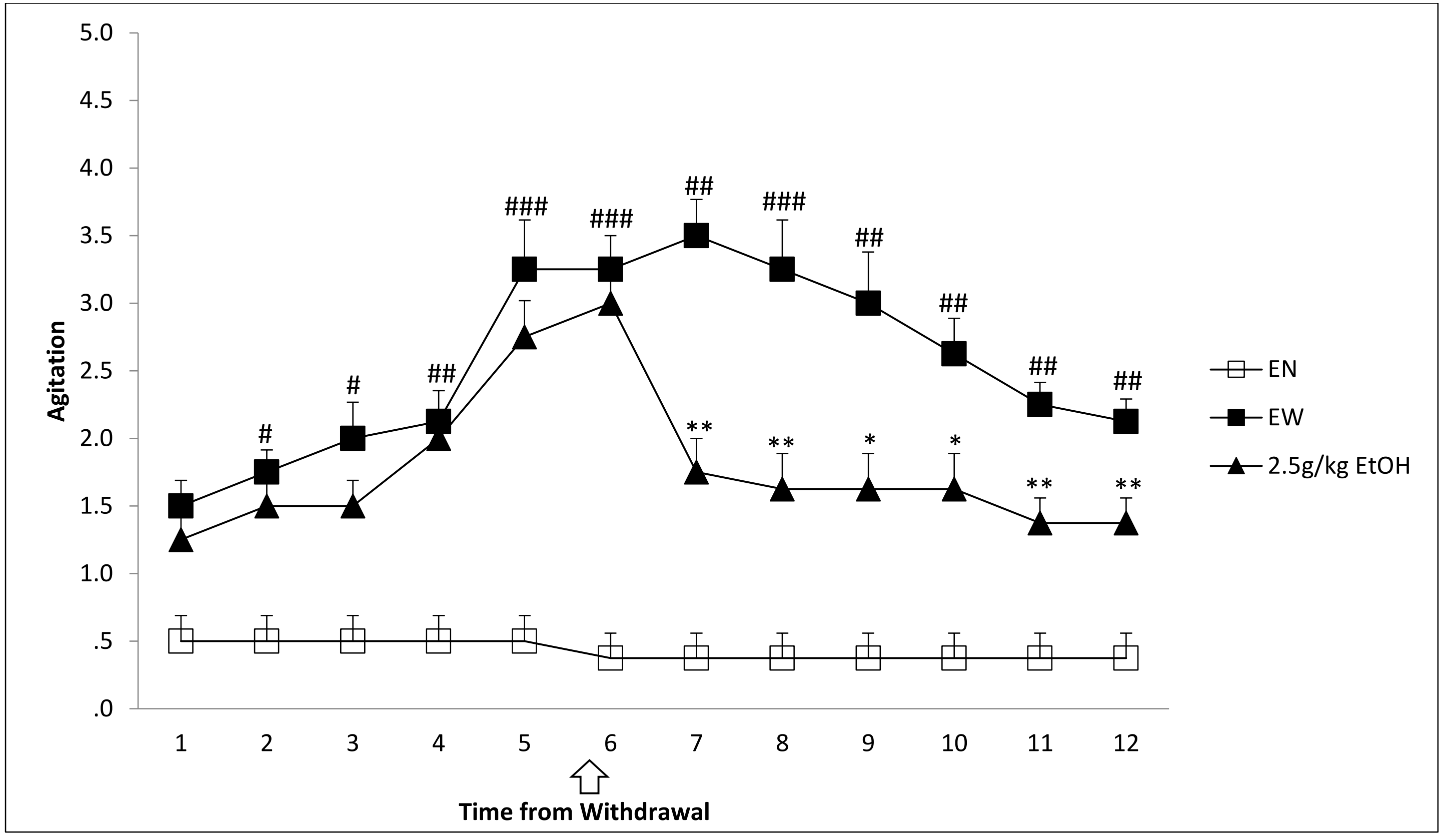

Supplementary Figure S4. Score for agitation during ethanol withdrawal.

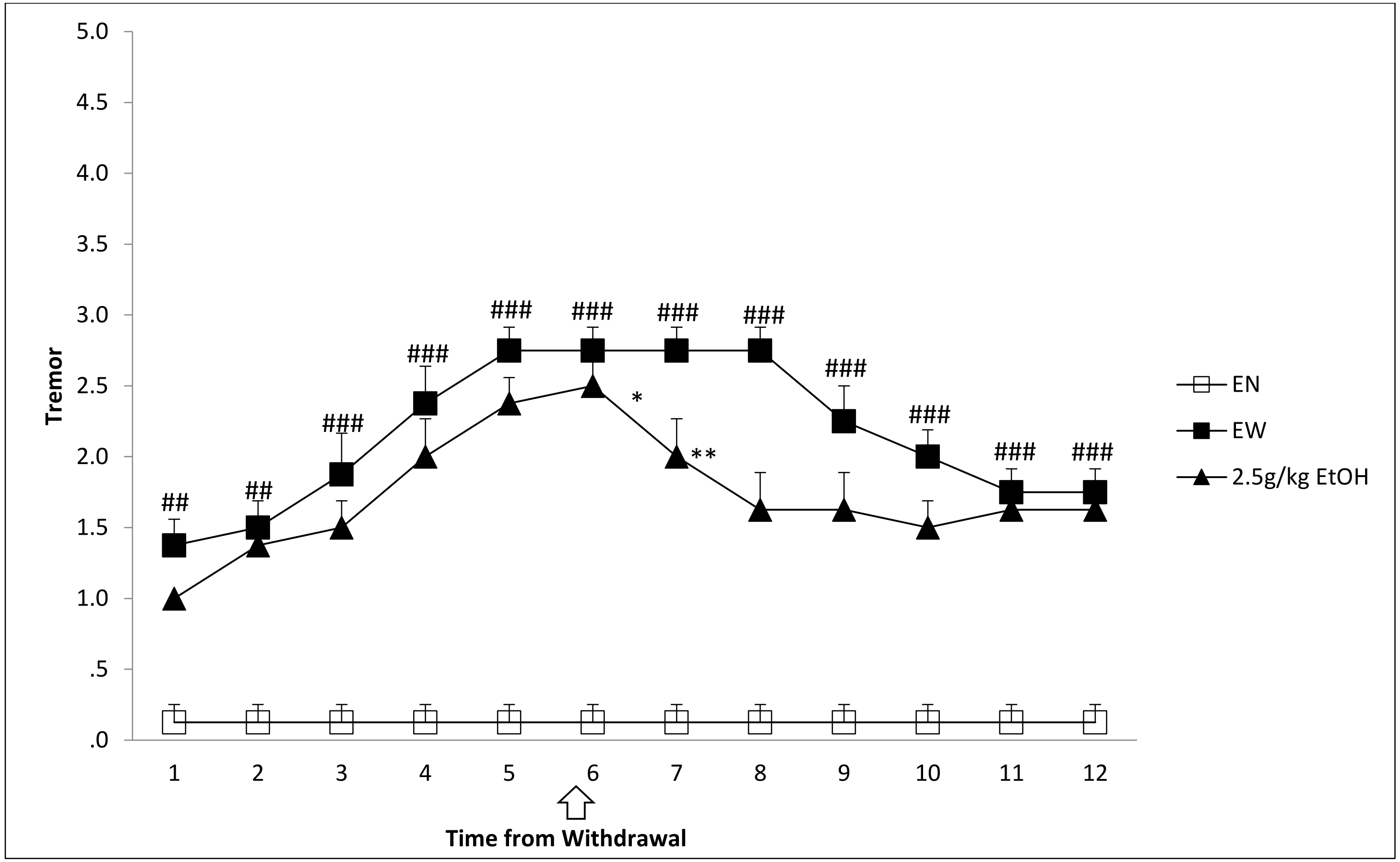

Supplementary Figure S5. Score for tremor during ethanol withdrawal.

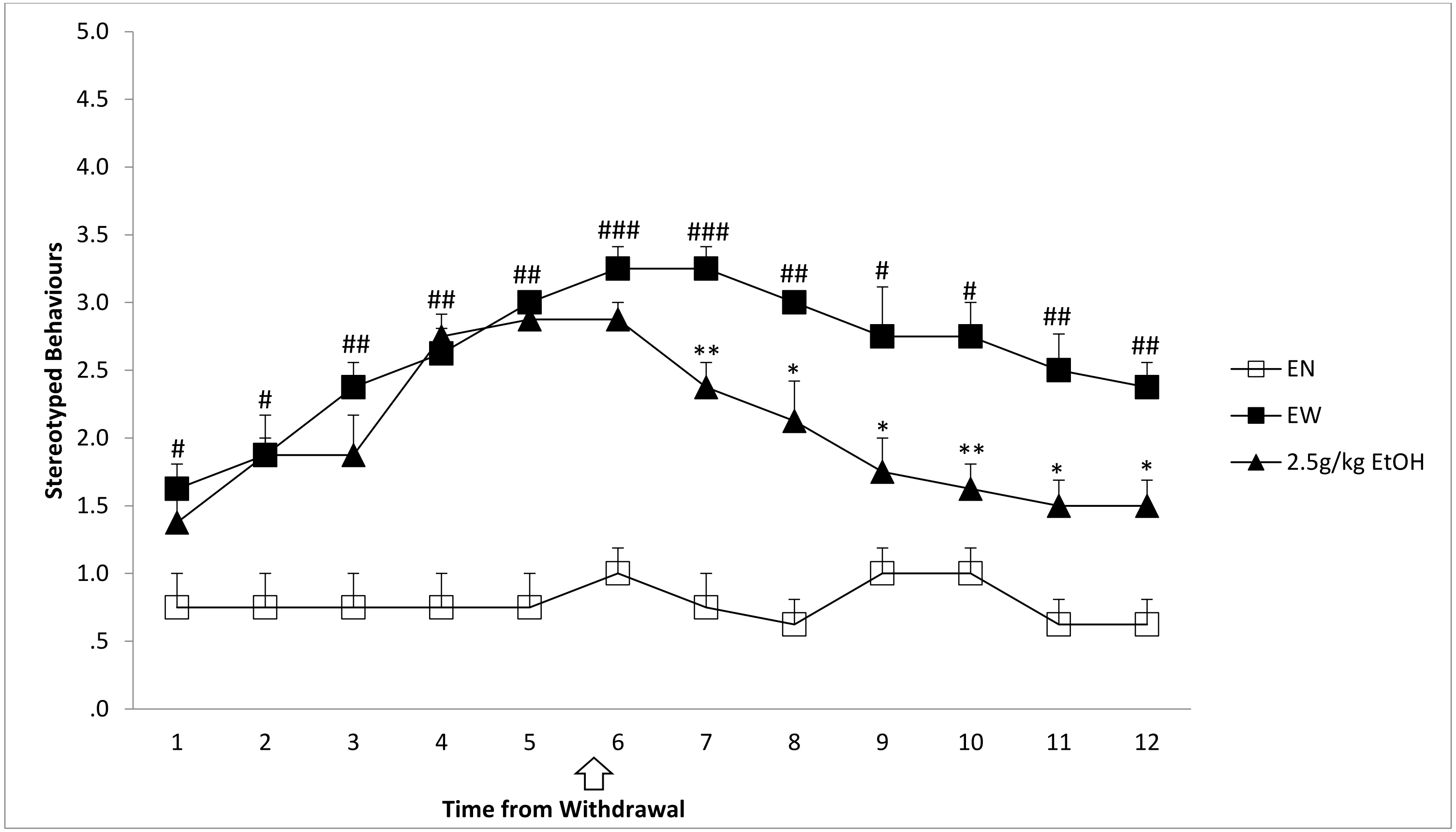

Supplementary Figure S6. Score for stereotyped behaviours during ethanol withdrawal.

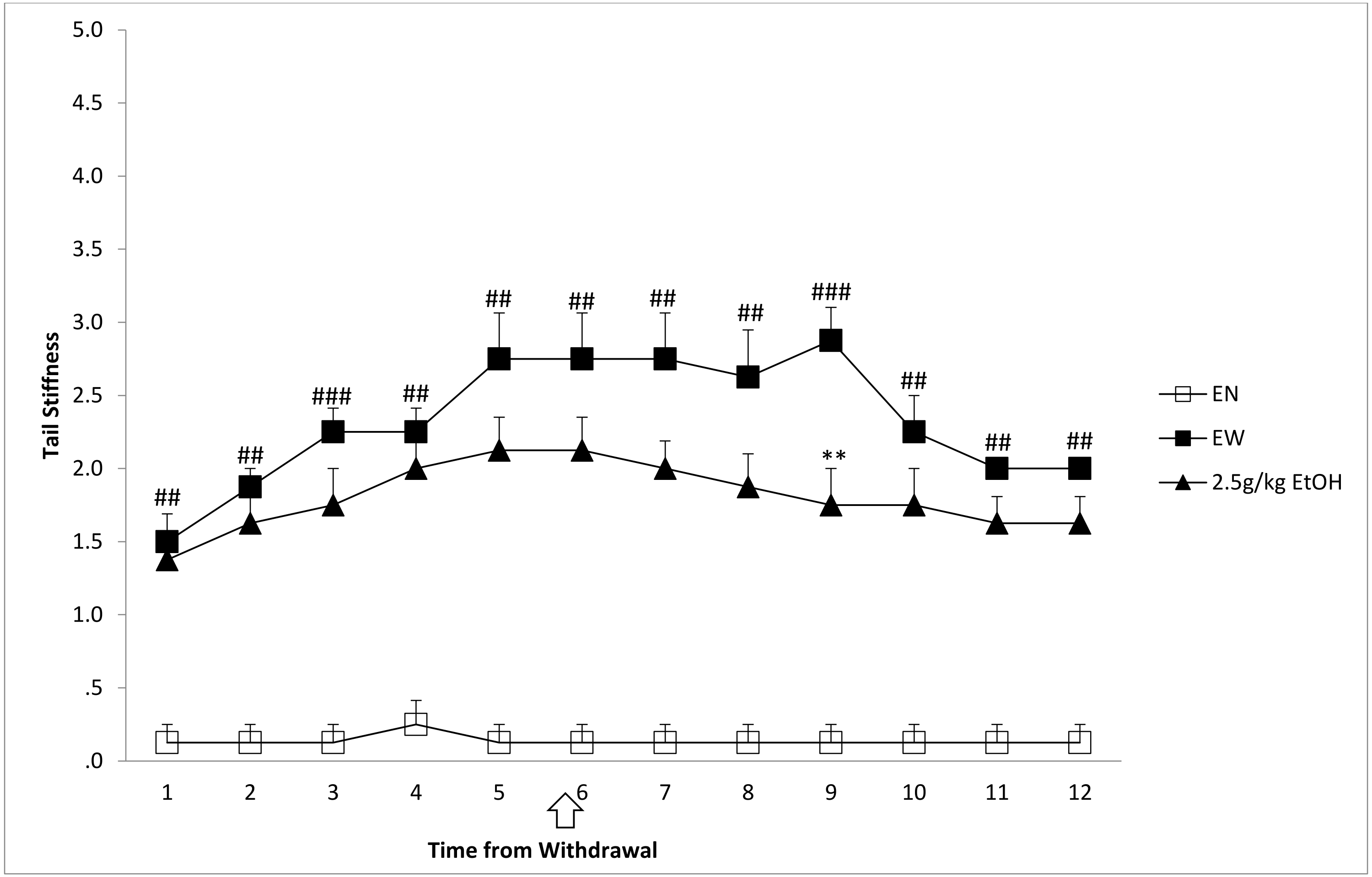

Supplementary Figure S7. Score for tail stiffness during ethanol withdrawal.

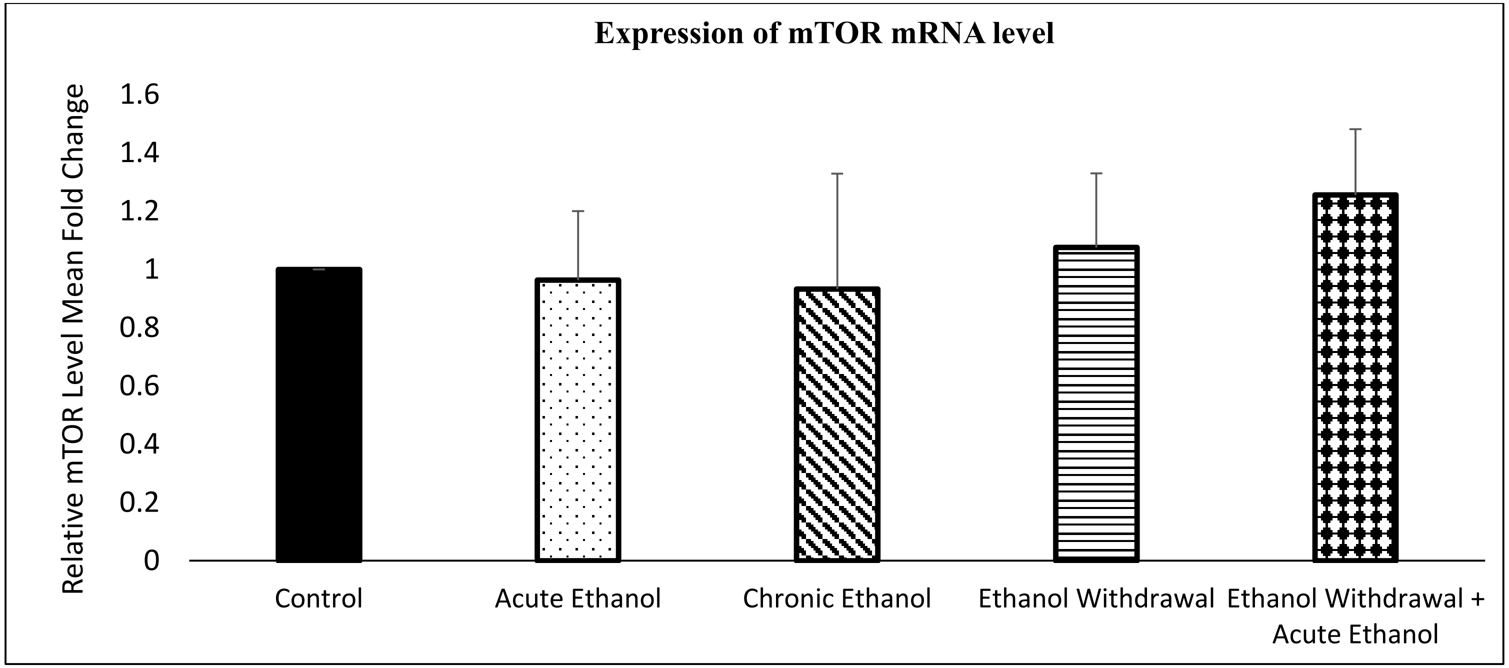

Supplementary Figure S8. Relative mTOR mRNA level in the amygdala of rats.

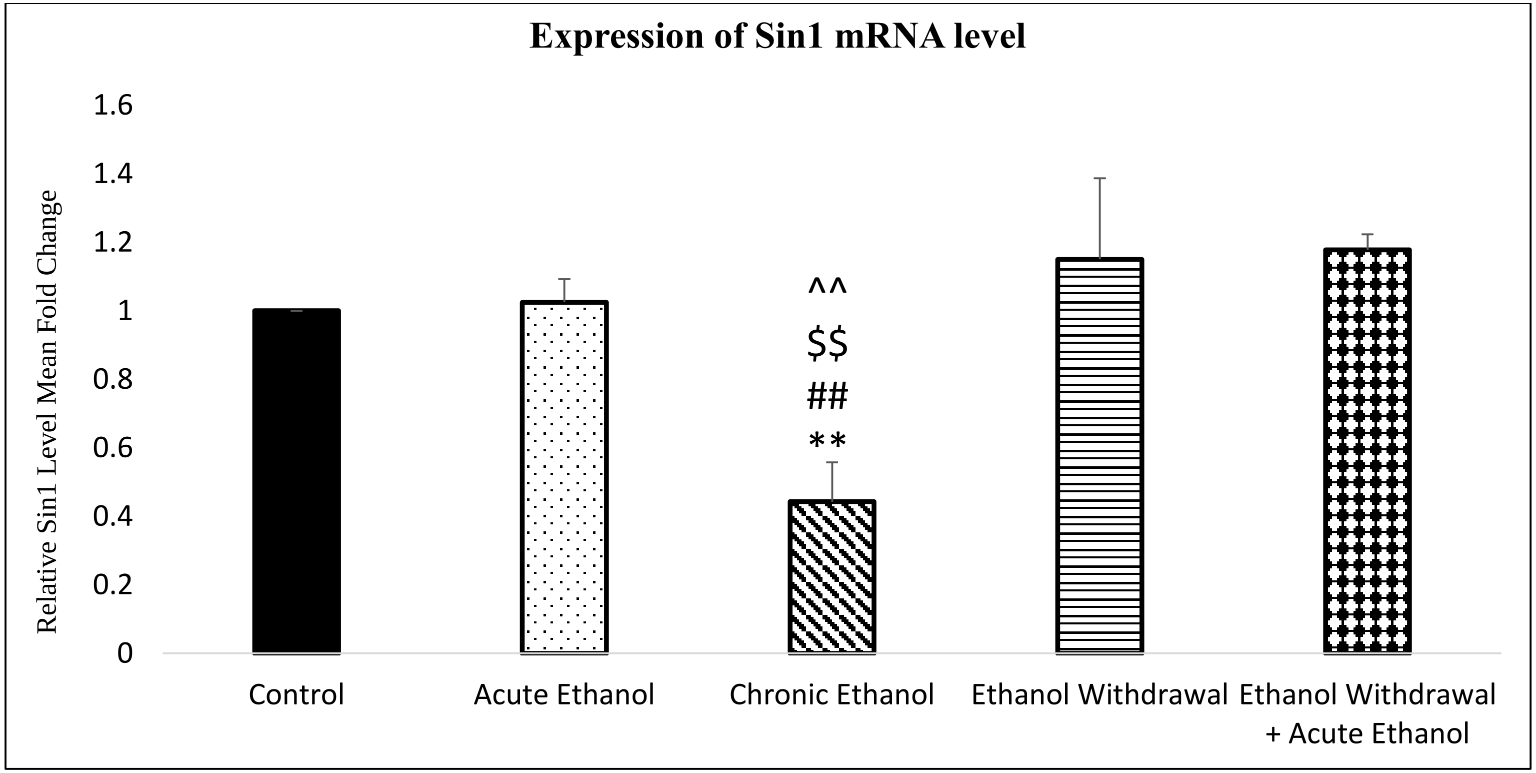

Supplementary Figure S9. Relative Sin1 mRNA level in the amygdala of rats.

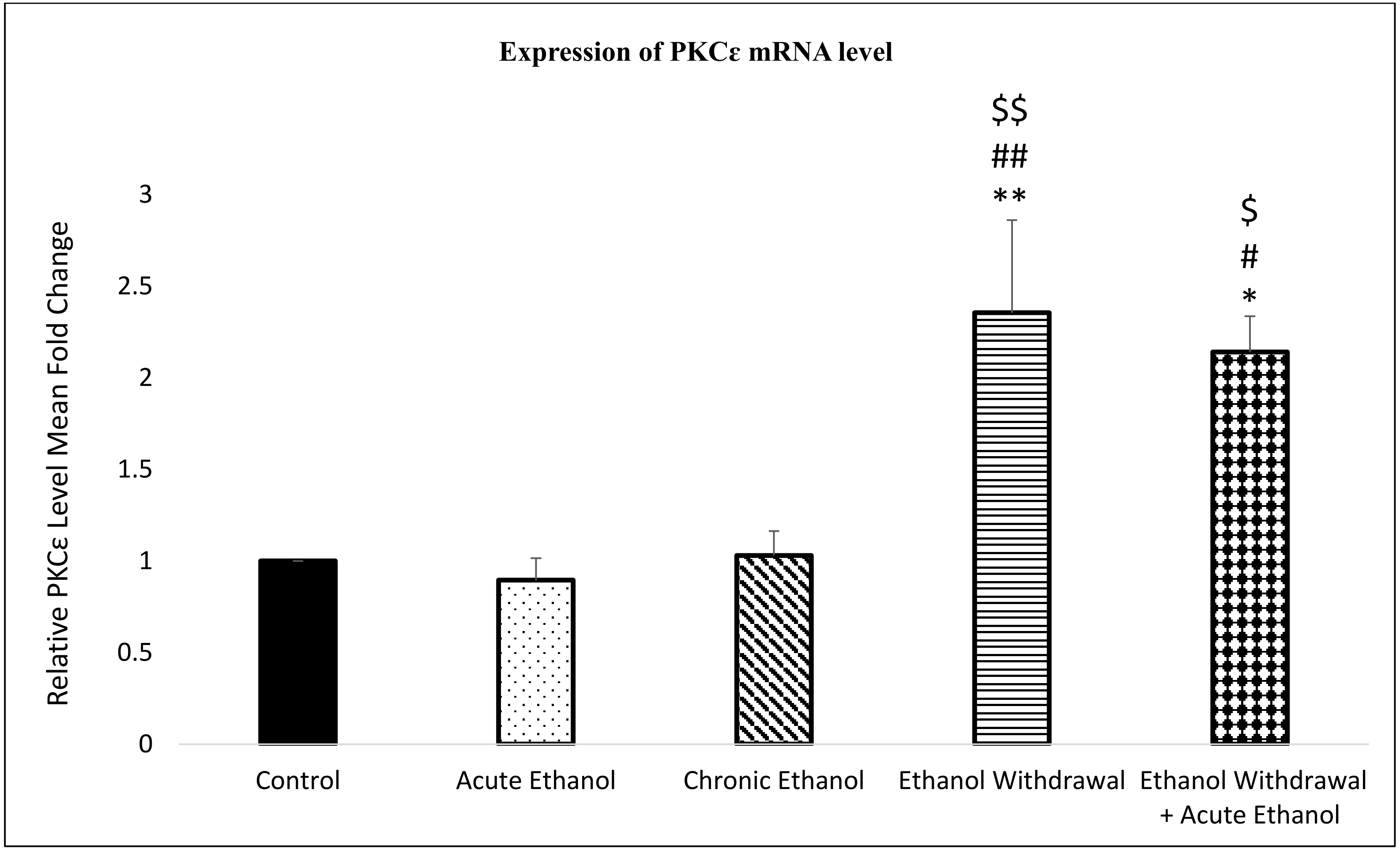

Supplementary Figure S10. Relative PKCε mRNA level in the amygdala of rats.

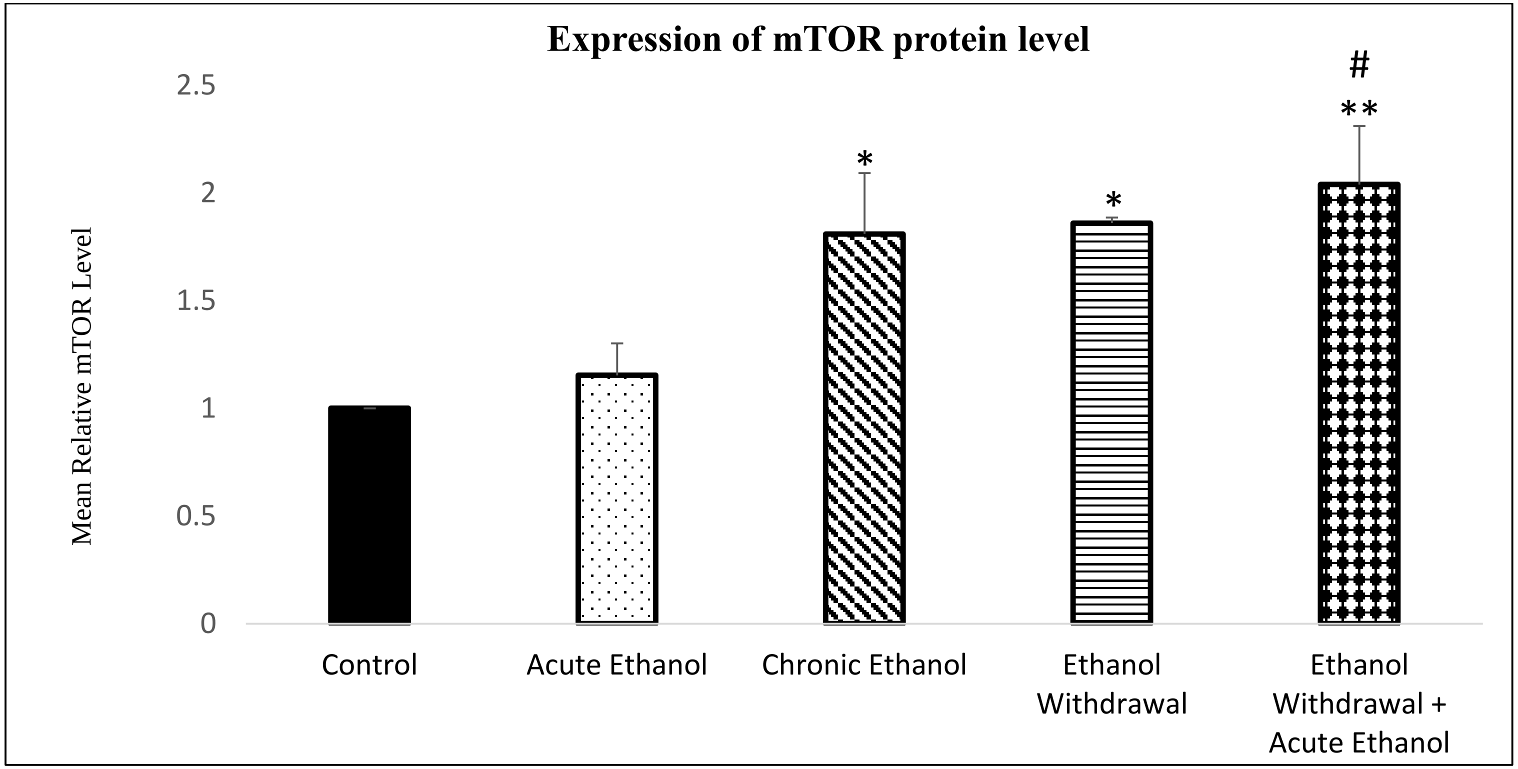

Supplementary Figure S11. The changes in protein expression of mTOR in whole cell lysate of amygdala.

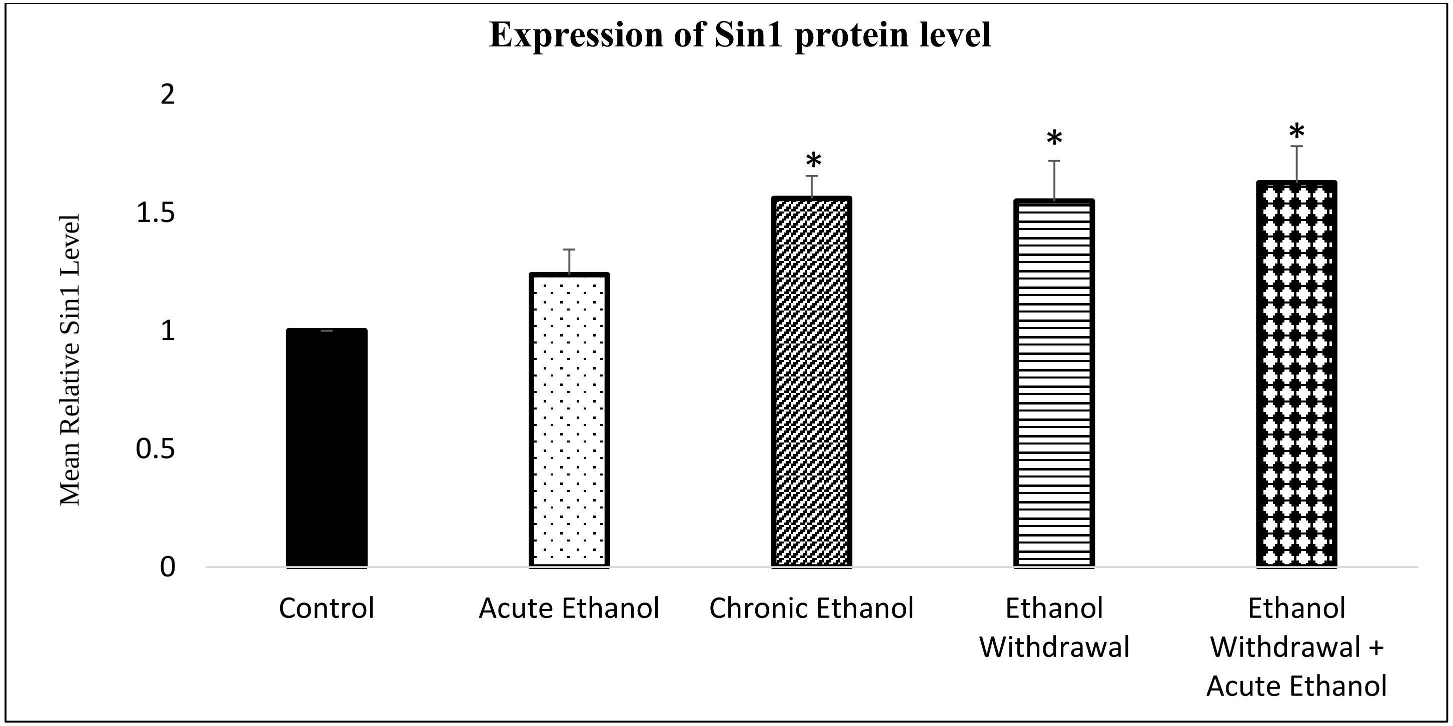

Supplementary Figure S12. The changes in protein expression of Sin1 in whole cell lysate of amygdala.

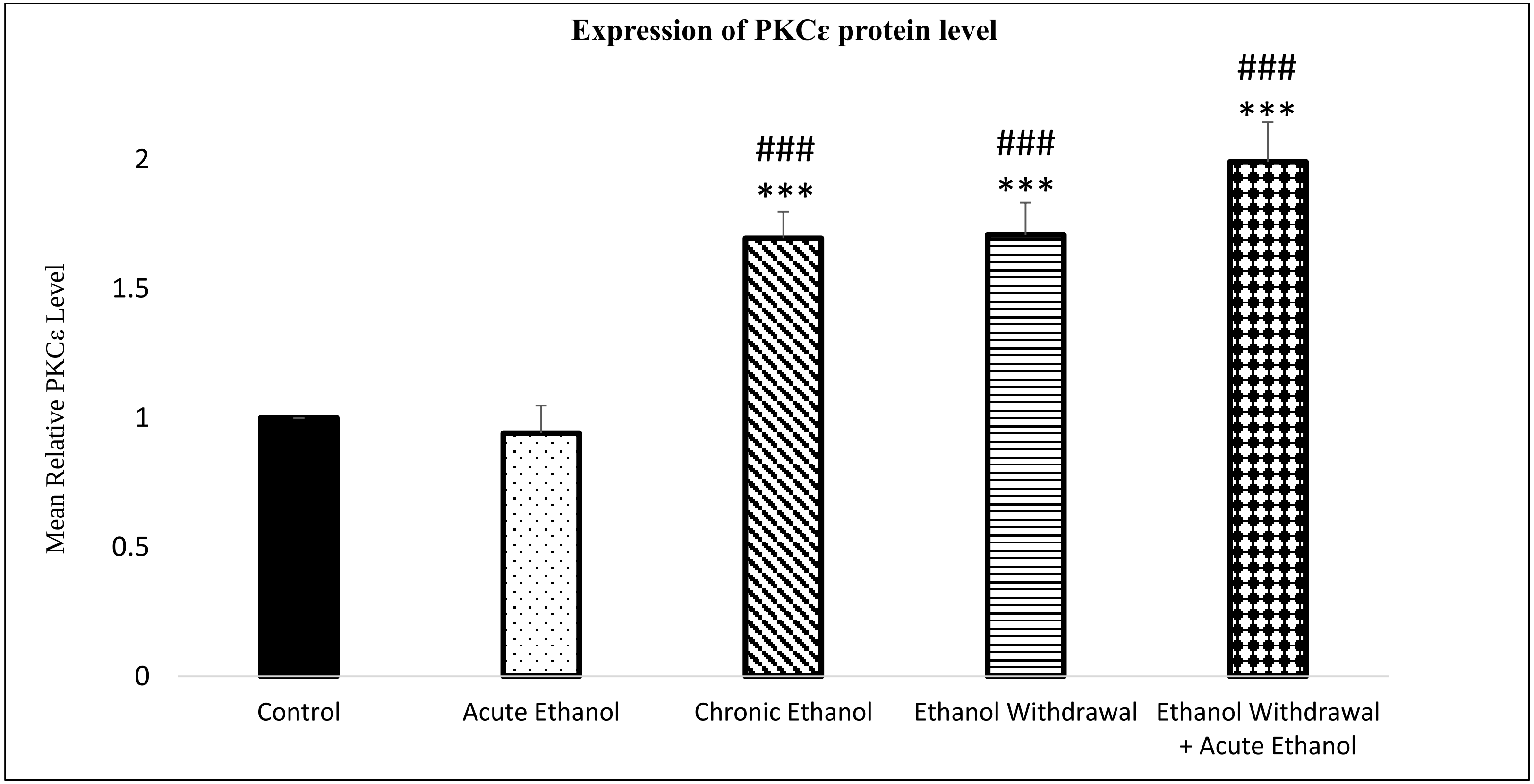

Supplementary Figure S13. The changes in protein expression of PKCε in whole cell lysate of amygdala.

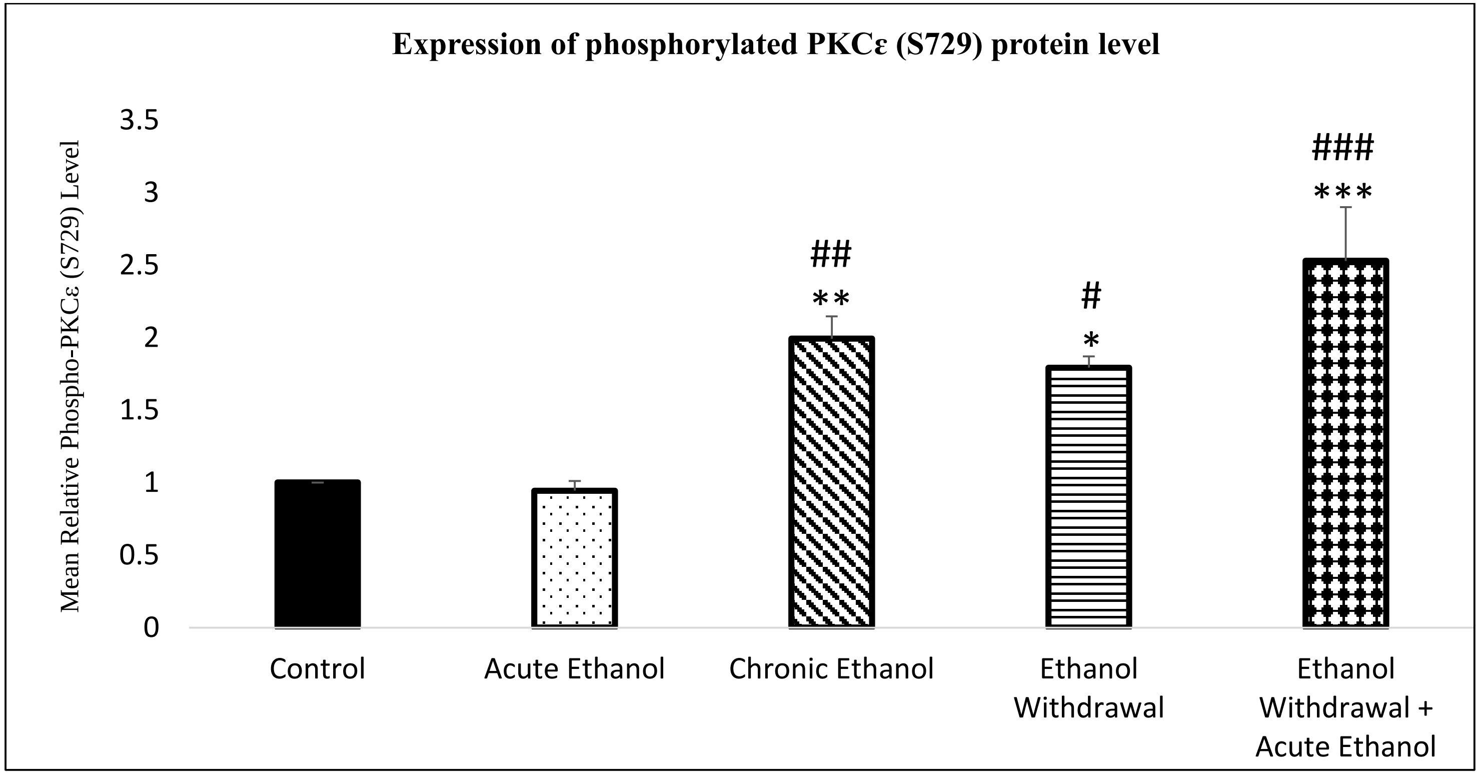

Supplementary Figure S14. The changes in protein expression of phosphorylated PKCε in whole cell lysate of amygdala.

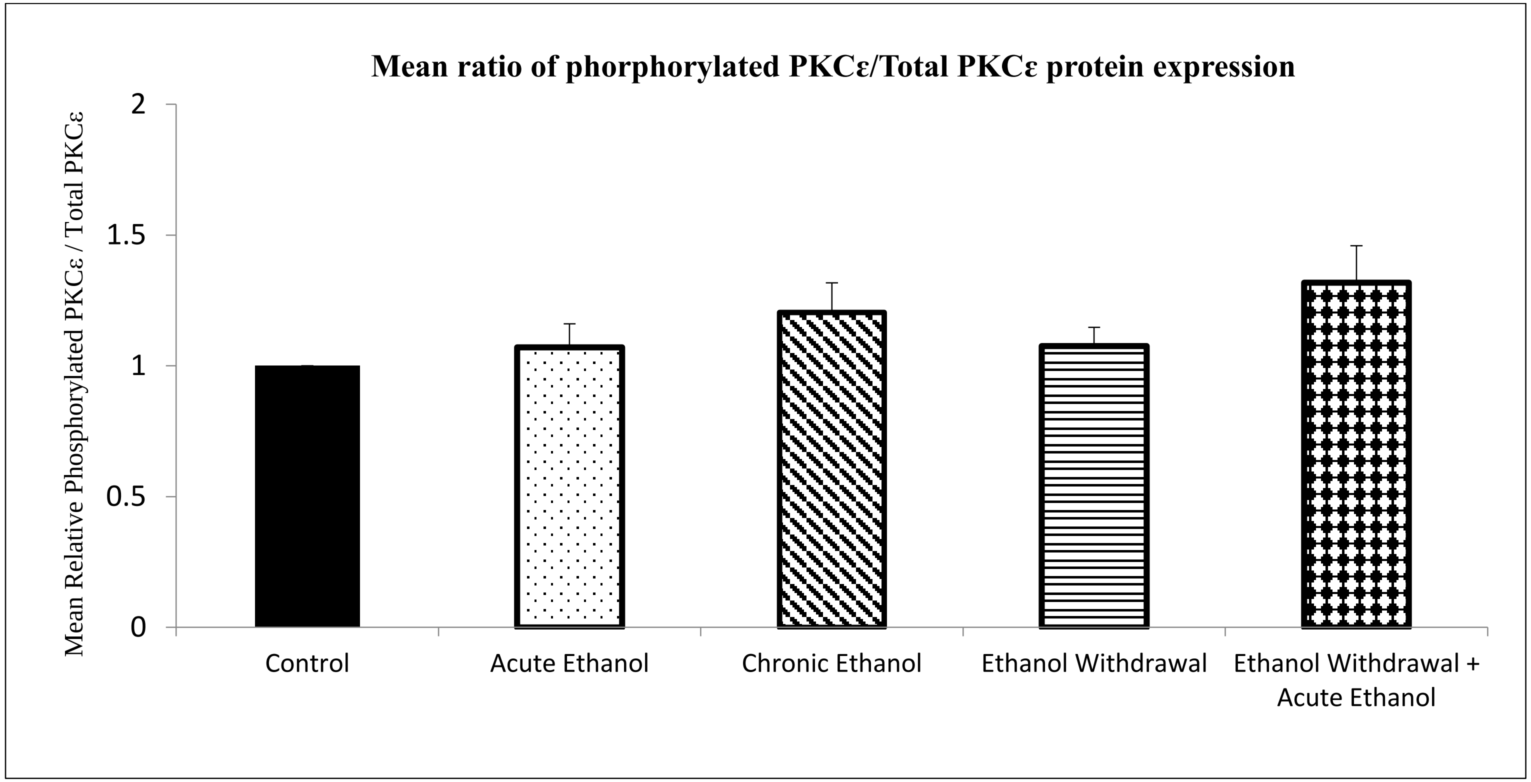

Supplementary Figure S15. Mean ratio of phosphorylated PKCε/Total PKCε protein expression in whole cell lysate of amygdala.
