# Supplementary material for: Alcohol Dependence Modulates Amygdalar mTORC2 and PKCε Expression in a Rodent Model
Source: Nutrients. 2023 Jul 5;15(13):3036. doi: 10.3390/nu15133036 (PMC10346598; doi:10.3390/nu15133036)
Supplement: Supplementary file 1 [file nutrients-15-03036-s001.zip › nutrients-2455939-supplementary Tables S1-S2.pdf]

Supplementary Table S1. Evaluation of EW symptoms

Behavioural signs	Score
Abnormal posture	0: Abnormal posture was absent<br>1: back hunched, mild head-down,<br>2: back hunched, moderate head-down,<br>3: back hunched, prominent head-down,<br>4: In addition, hind legs wide apart<br>5: In addition, fore limbs apart
Abnormal gait	0: Abnormal gait was absent<br>1–2: rearing was normal with mild difficulty in ambulating<br>3–4: rearing was normal with moderate difficulty in ambulating<br>5: no presence of rearing with prominent difficulty in ambulating
Agitation	0: Aggressive behavior or irritability were absent<br>1: mild or moderate irritability were shown by rats<br>2: highly irritable<br>3: moderately aggressive and handling vocalization were present<br>4: highly aggressive and handling vocalization were present<br>5: highly aggressive and spontaneous vocalization were present

Tail stiffness	0: Tail rigidity was absent<br><br>1: mild rigidity of tail<br><br>2: moderate rigidity of tail<br><br>3: tail was rigid but during ambulation was mildly flexible<br><br>4: tail was rigid and during ambulation was not flexible<br><br>5: tail very rigid and not flexible during ambulation
Stereotyped behavior	0: stereotyped behavior was absent<br><br>1: only one stereotyped behavior was present<br><br>2: two stereotyped behaviors were present<br><br>3: three stereotyped behaviors were present<br><br>4: four stereotyped behaviors were present<br><br>5: all stereotyped behaviors were present<br><br>During ethanol withdrawal, stereotyped behaviors are considered including grooming,head-waving, sniffing,chewing, and gnawing.
Tremor	Rats were lifted vertically by the tail to measure the tremor; rats with clearly discernible forelimb tremor when rotated 180 degrees around the axis of the tail were given a positive score.<br>0: No extension of forelimb and tremor<br><br>1: No display forelimb extension but clear distinct forelimb tremor when lifted by the tail and rotated 180 degree.<br><br>2: Rats showing the aforementioned behavioral change only when they were rotated 180 degree around the axis of the tail.<br><br>3: Immediate forelimb extension and violent generalized forelimb whole body tremor.

Supplementary Table S2. Characteristics of the pre-designed gene specific primer used in the present study

Gene	Gene ID	RefSeq ID	Exons	Primer sequence (5′-3′)
Prkce	29340	NM_017171	8-10	GACCAGGAACTAAAAGAACTTG (Forward)<br>CTTTGCCTAACACCTTGATG (Reverse)
Mtor	56718	NM_019906	8-10	AGAAATTTGATCAGGTGTGC (Forward)<br>TTCCTTTTCCTTCTTGACAC (Reverse)
Mapkap1	296648	NM_001011964	9-10	CAGCCACCATTATAAGTCATTC (Forward)<br>ACTTTGTCTCCAGAGATACC (Reverse)
Actb	81822	NM_031144	Within single exon	AAGACCTCTATGCCAACAC (Forward)<br>TGATCTTCATGGTGCTAGG (Reverse)
