# Supplementary figures and images for: Alcohol Dependence Modulates Amygdalar mTORC2 and PKCε Expression in a Rodent Model
Source: Nutrients. 2023 Jul 5;15(13):3036. doi: 10.3390/nu15133036 (PMC10346598; doi:10.3390/nu15133036)

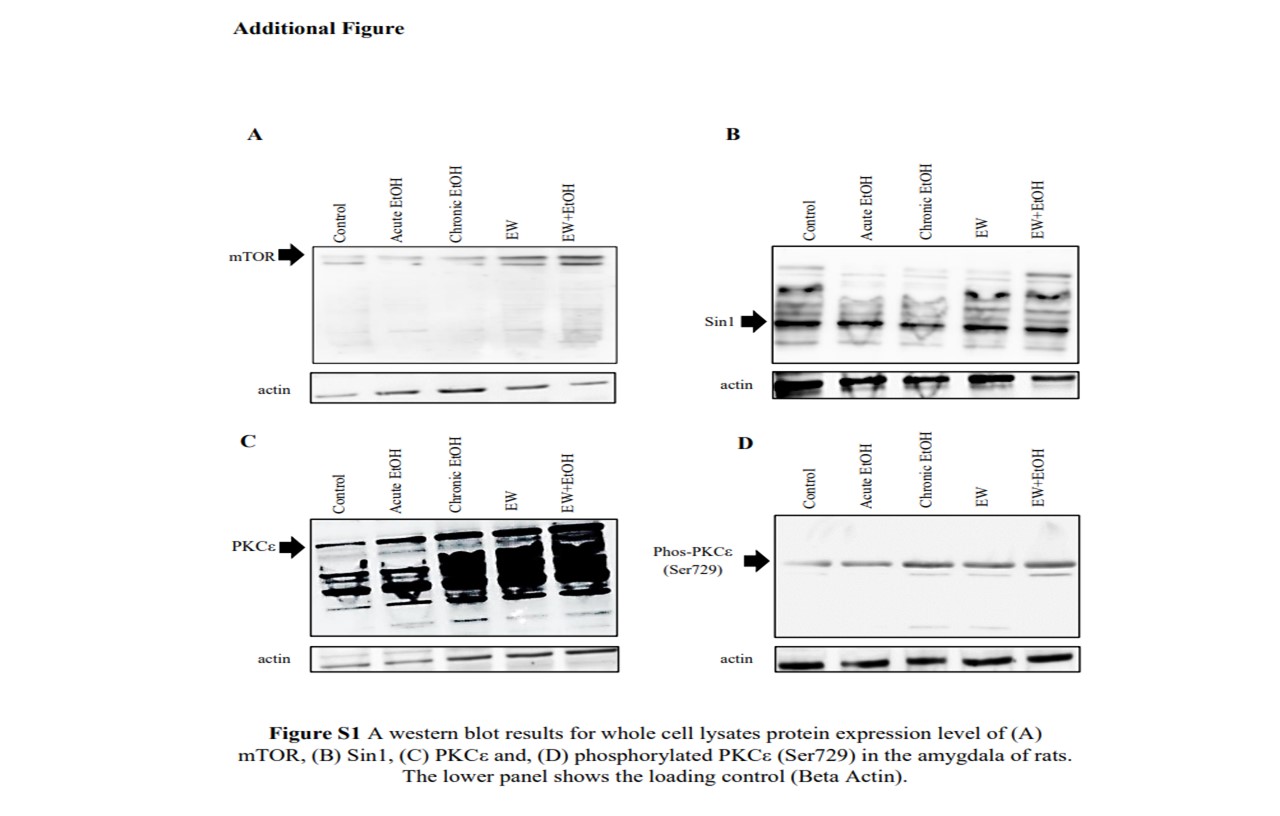

Supplement: Supplementary file 1 [file nutrients-15-03036-s001.zip › nutrients-2455939-supplementary Figure S1.jpg]
